# Cyclic Oxidation of Titanium Grade 2

**DOI:** 10.3390/ma13235431

**Published:** 2020-11-28

**Authors:** Krzysztof Aniołek, Adrian Barylski, Marian Kupka, Grzegorz Dercz

**Affiliations:** Institute of Materials Engineering, University of Silesia, ul. 75 Pułku Piechoty 1A, 41-500 Chorzów, Poland; adrian.barylski@us.edu.pl (A.B.); marian.kupka@us.edu.pl (M.K.); grzegorz.dercz@us.edu.pl (G.D.)

**Keywords:** cyclic oxidation, morphology, oxidation kinetics, oxide scale, titanium Grade 2

## Abstract

This paper presents the results of research into the cyclic oxidation of titanium Grade 2. The value of titanium Grade 2 oxidation activation energy was determined based on an analysis of the Arrhenius diagram. The result was 205.3 kJ/mol. After cyclic oxidation at a temperature of 600 °C, the presence of oxides in an acicular system was observed on the surface. The specimen surface after oxidation at 650 °C was characterised by the presence of fine oxide particles, while after oxidation at 700 °C, the obtained oxide layer was composed of large oxide particles. The layers obtained after oxidation at 600 °C had the lowest thickness (1.26 and 2.12 µm), while those obtained at 700 °C had the highest thickness (5.17 and 9.45 µm). Examination of the phase composition after cyclic oxidation showed that the oxide layers obtained at temperatures of 600, 650 and 700 °C were composed of TiO_2_ (rutile) only. No presence of other phases was found. The oxide layers formed in the cyclic oxidation process were characterised by different thicknesses, depending on the oxidation parameters. It was found that cyclic oxidation contributed to a considerable increase in the surface hardness of titanium Grade 2.

## 1. Introduction

Titanium and its alloys belong to a group of metallic materials widely used in industrial areas and in the biomedical sector. Their wide application results from a good combination of mechanical properties, low density, excellent corrosion resistance and the best, compared to other metallic biomaterials, biocompatibility [1,2,3,4,5,6].

Due to their unique mechanical and physical properties, as well as their high corrosion resistance, titanium and its alloys can be found in engineering and biomedical applications. They also play an important role when the weight of the structure and its strength are decisive. For these reasons, titanium and its alloys are widely used, e.g., in aviation, space industry and in the production of sports equipment [7,8,9,10]. Due to their good corrosion resistance, these materials are also used in the chemical, marine, automotive and military industries [11]. Titanium and its alloys also have increasing use in medicine [12,13]. Their usefulness in the biomedical industry is determined by their very good corrosion resistance in the tissue environment and the associated best among metallic materials biocompatibility [14,15,16,17].

The analysis of previous scientific papers shows that the optimization of biological, mechanical and tribological properties of titanium and its alloys has already been achieved through the development of optimal chemical and phase composition as well as methods of plastic working and heat treatment [18]. In order to obtain the best possible application results, it is necessary to implement the achievements of material engineering, including surface engineering, which is conducive to the generation of new, in terms of quality, technical solutions and improves the reliability of implants made of traditional metallic biomaterials [19,20]. The most commonly used methods are anodising, ion implantation, laser machining, plasma spraying, PVD (Physical Vapor Deposition) and CVD (Chemical Vapor Deposition) [21,22,23,24]. Apart from the above-mentioned methods, the natural phenomenon of high affinity of titanium and its alloys to oxygen can be taken advantage of. By forming oxide scales on the surface of titanium and its alloys, it is possible to obtain an increase in resistance to biological corrosion, biocompatibility, biological activity, surface topography, as well as mechanical and tribological properties. One of the most effective methods of using the low resistance to oxidation of titanium and its alloys is thermal oxidation, which was widely studied in particular on the Ti-6Al-4V alloy [25,26,27,28,29]. A variation of this method is cyclic oxidation, which, however, has not been studied as extensively and systematically as thermal oxidation. The process of cyclic oxidation, although more difficult to implement, may bring even more measurable benefits in comparison with thermal oxidation. The current state of knowledge regarding the influence of cyclic oxidation parameters on the properties of the obtained oxide scales should be considered insufficient. Analysis of the literature in this scope has shown that titanium Grade 2 has never been subjected to cyclic oxidation. Only a few papers devoted to cyclic oxidation of alloy Ti-6Al-4V can be found in the literature [30,31,32]. It is an extremely important challenge to find the optimal temperature and time conditions, so that the obtained layers could have the required thickness while ensuring their appropriate adhesion.

The results of the research presented in this paper focus on the process of cyclic oxidation of titanium Grade 2 in terms of achieving the best functional characteristics of the obtained oxide scales. In this paper, the oxidation kinetics, morphology, phase composition and mechanical properties of oxide scales produced by cyclic oxidation on Grade 2 titanium are compared.

## 2. Materials and Methods

The material used for tests was titanium Grade 2 in the form of rods. The tests were carried out on specimens in the form of 2 mm thick discs, 12 mm in diameter. Analysis of the chemical composition of titanium Grade 2 was made using the following research methods: ICP-OES (Inductively Coupled Plasma Optical Emission Spectrometry), HFIR and high-temperature extraction (for testing gas content).

Microstructural tests of titanium Grade 2 in its initial state were performed on an Olympus GX-51 optical microscope (Tokyo, Japan). Microscopic observations were carried out at a magnification of 500×. The grain size of titanium Grade 2 was determined by means of the Olympus Stream Essentials.

Before proceeding to tests, the specimen surfaces had been ground using 600, 1200 and, finally, 2000 grit abrasive paper, after which they were polished with diamond pastes. In the next phase, the specimens were degreased in acetone. The cyclic oxidation process was carried out in a chamber furnace of the Czylok company, (Jastrzębie-Zdrój, Poland) in the air atmosphere, at temperatures of 600, 650 and 700 °C. For each temperature variant, oxidation was carried out in 12 cycles. Soaking time for 1 cycle was 6 h, so the total oxidation time for 12 cycles was 72 h. After the specified soaking time in a given cycle, the specimens were removed from the furnace and cooled in the air to ambient temperature. To describe the oxidation kinetics, a gravimetric method was used. The tested specimens were weighed after each oxidation cycle on a laboratory microbalance.

To characterize the course of cyclic oxidation of titanium, the following dependence was used:

(1) $${\left(\frac{\Delta W}{A}\right)}^{n}=kt$$

where Δ*W*—increase in specimen mass, *A*—specimen surface area, *t*—oxidation time, *k*—exponential constant of oxidation rate.

The values of constant *K_p_* were determined in the tests from the slope of the straight line adjusted by using the linear regression method to dependence (Δ*W*/*A*)^2^ with respect to time *t*. Next, the cyclic oxidation activation energy was determined from the following equation:

(2) $$K_{p}=K_{0}exp^{-\left(\frac{Q}{RT}\right)}$$

where *Q—*activation energy, *K*_0_—pre-exponential factor, *R*—gas constant (8.3144 Jmol-1K-1), *T*—absolute temperature.

Examination of the morphological features of oxides formed as a result of cyclic oxidation was conducted using a JEOL JSM-6480 electron microscope (Tokyo, Japan). Microscopic images were recorded at a magnification of 5000× after 4 and 12 oxidation cycles at temperatures of 600, 650 and 700 °C. The same equipment was to measure thickness of the produced layers at a magnification of 2000×. The process of specimen preparation for the observation of oxide scales on cross-sections consisted of several phases. In the first phase, the specimens were cut into 2 parts and then they were positioned with oxide layers adjacent to each other and mounted. In the next phase, the microsections were ground and polished in accordance with the methodology described above.

X-ray diffraction examination was performed using an X’Pert PW3040/60 instrument (Almelo, The Netherlands). The X-ray tube was supplied with voltage U = 40 kV and current I = 30 mA. The length of the X-ray radiation wave emitted by the copper tube was 1.54178 Å. Tests were conducted at constant incident angles of α = 0.15°; 0.25°; 0.50°; 1.00° and 1.50°. The counter moved within the angular range from 20° to 80° 2*θ* with a measuring step of 0.05° 2*θ*.

The hardness of the oxide scales was examined using a Vickers microhardness tester (model 401MVD, Wolpert Wilson, Worcester, MA, USA). The measurements were performed for a range of loads from 25 to 200 gf (245–1960 mN). The load was held during the measurement for 15 s. For each specimen, 40 indents were made (10 for one load variant). The paper presents averaged results of the measurements. The measurements were performed on a non-oxidized surface and after cyclic oxidation at 600, 650 and 700 °C.

## 3. Results and Discussion

### 3.1. Characteristics of Research Material

**Figure 1:**
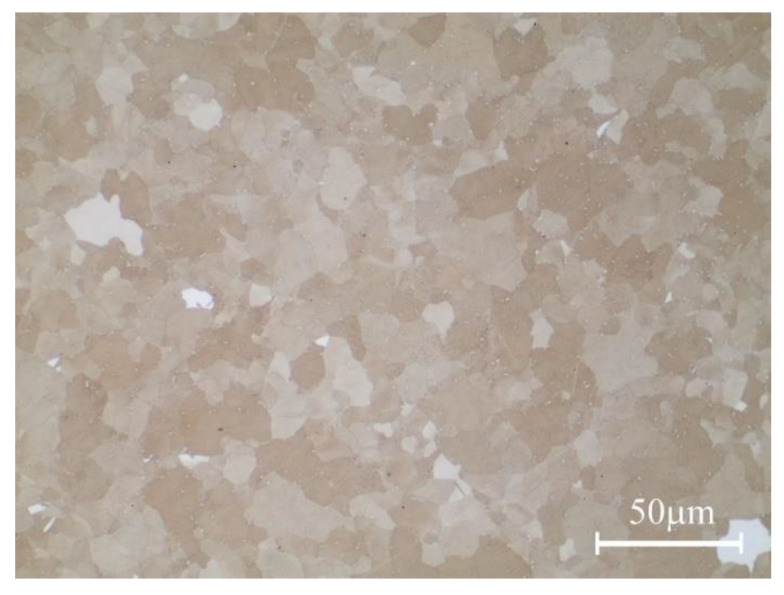
Microstructure of titanium Grade 2.

**Figure 2:**
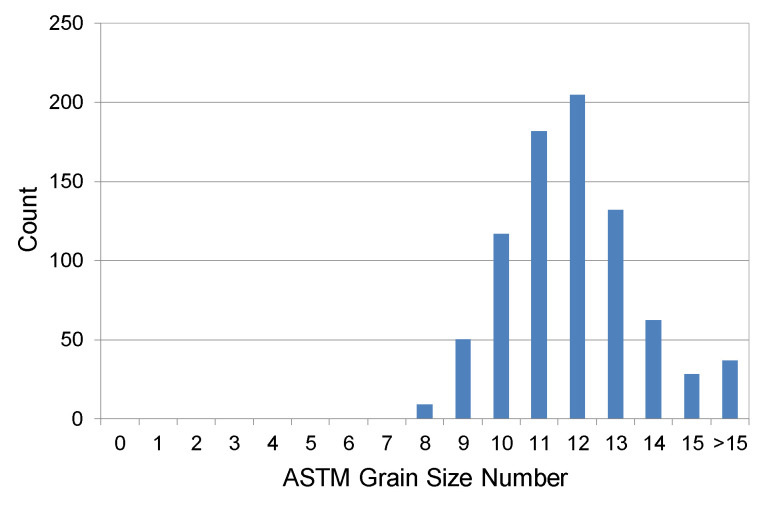
Titanium Grade 2 grain size distribution according to the ASTM standard.

The material used for the tests was titanium Grade 2 manufactured by S-Tech Corporation. The microstructure and chemical composition of the material are presented in Figure 1 and in Table 1. Detailed parameters of the grain size are provided in Table 2 and in Figure 2.

Microscopic observations and a quantitative analysis of the microstructure showed that titanium Grade 2 had a granular structure with the grain size 11 on the ASTM scale. The slightly increased oxygen content (0.18%) is considered to be an alloying agent in this case, as it distinctly increases the strength properties of the material. In this case, the increase in strength is caused by α solution strengthening. The chemical composition of the material for tests was in compliance with the certificate delivered by its manufacturer.

### 3.2. Kinetics of Cyclic Oxidation of Titanium Grade 2

**Figure 3:**
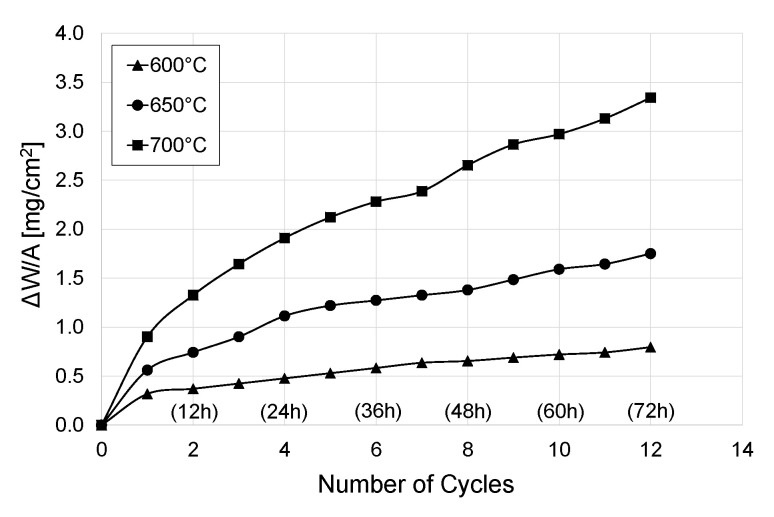
Kinetics of cyclic oxidation of titanium Grade 2 at temperatures of 600, 650 and 700 °C, depending on the number of cycles (1 cycle = 6 h).

**Figure 4:**
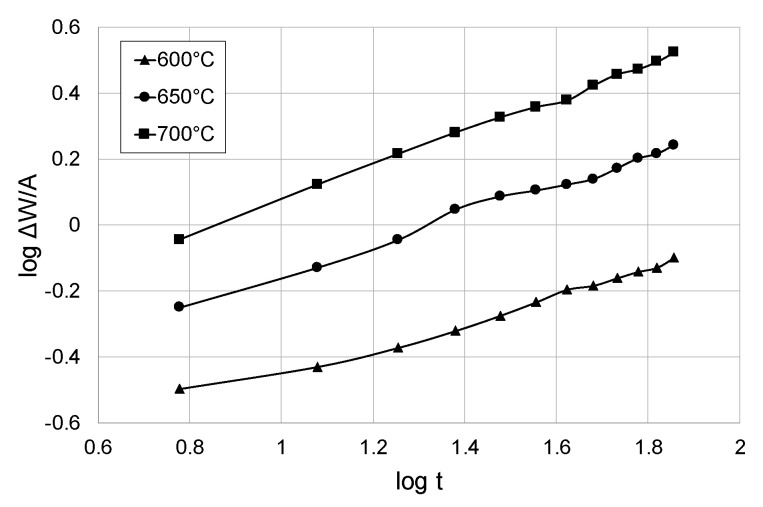
Graph showing the dependence of log Δ*W*/*A* on log t.

**Figure 5:**
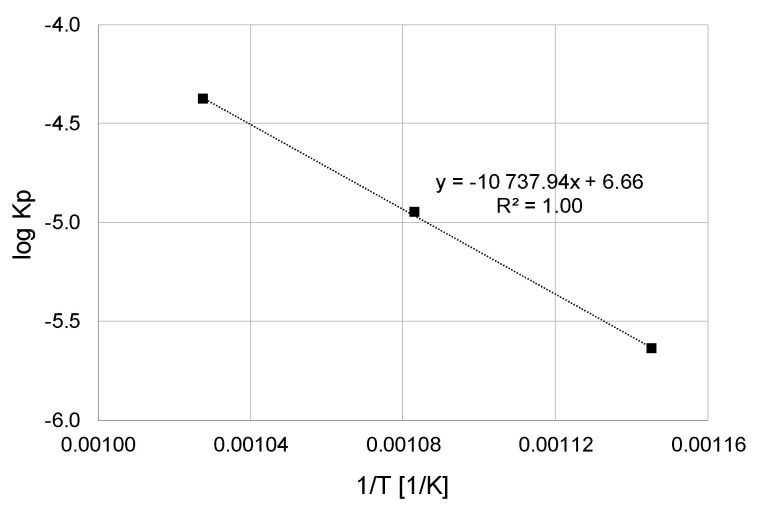
Arrhenius graph for titanium Grade 2 after cyclic oxidation at 600–700 °C for 72 h (12 cycles).

The examination results of the kinetics of cyclic oxidation of titanium Grade 2 after oxidation at 600, 650 and 700 °C are presented in Figure 3, Figure 4 and Figure 5.

On the basis of the obtained results, it was found that the basic oxidation parameters, such as temperature and number of cycles, have a significant influence on the intensity of cyclic oxidation of titanium Grade 2. The study shows that the lowest mass gain occurred after oxidation at the temperature of 600 °C. After 12 oxidation cycles, the gain of sample mass was insignificant and amounted to 0.80 mg/cm^2^. An increase in oxidation temperature to 650 °C caused an increase in oxidation intensity. In this temperature variant, the mass gain (1.75 mg/cm^2^) was more than twice as high as at 600 °C. However, the highest intensity of the process was observed after cyclic oxidation at the temperature of 700 °C. In this case, the mass gain (3.34 mg/cm^2^) was almost 4 times higher than in the case where the oxidation temperature was 600 °C.

For similar temperature/time parameters of isothermal oxidation presented in paper [33] it was found that the cyclic oxidation process was characterised by higher intensity, and thus allowed obtaining a much higher increase of oxide scale. After cyclic oxidation at the temperature of 600 °C, the gain in mass was by approx. 50% higher in comparison with the traditional method of thermal oxidation. It was also found that after cyclic oxidation at 700 °C, the gain in mass was by approx. 80% higher when compared to isothermal oxidation [33].

Figure 4 shows the dependence of log Δ*W*/*A* on log t. Next, the linear regression method was used to determine the value of exponent *n*. It was noticed that the oxidation proceeded in accordance with the parabolic law (approximately *n* = 2). At the next stage, values of the parabolic rate constant, K_p_, for oxidation of titanium Grade 2 were determined using the linear regression method. The obtained values are presented in Table 3.

Based on the calculations, it was found that the parabolic constant of the oxidation rate, K_p_, increased with the oxidation temperature. Increasing the oxidation temperature from 600 to 650 °C caused a nearly fivefold increase in the value of K_p_. At the same time, increasing the oxidation temperature from 650 to 700 °C caused the K_p_ value to increase by almost four times. When comparing the values of K_p_ constants for pure titanium, as obtained in paper [33], it was found during isothermal oxidation that much higher values of this parameter were obtained as a result of cyclic oxidation. In the case of cyclic oxidation, the value of K_p_ after oxidation at 600 °C was by more than twice higher, while at a temperature of 700 °C, it was higher by more than three times compared to isothermal oxidation.

Next, the values of constant K_p_ calculated in Table 1 were correlated with temperature using Arrhenius equation. The dependence log K_p_ − 1/T for cyclic oxidation at temperatures of 600, 650 and 700 °C is presented in Figure 5.

Based on an analysis of the obtained graph, the value of activation energy of titanium Grade 2 cyclic oxidation was determined as 205.3 kJ/mol. A comparable activation energy value of 215 kJ/mol was obtained in the study [34] for the range of isothermal oxidation temperature of 700–950 °C. In the case of similar parameters of isothermal oxidation presented in paper [33], a higher value of activation energy of the oxidation process of 278 kJ/mol was obtained. However, the differences in the obtained value for cyclic and isothermal oxidation may be due to the fact that the calculations of activation energy were performed in a slightly different temperature range (600–700 °C for cyclic oxidation, and 600–800 °C for thermal oxidation).

### 3.3. Examination of the Morphological Features of the Produced Oxides

**Figure 6:**
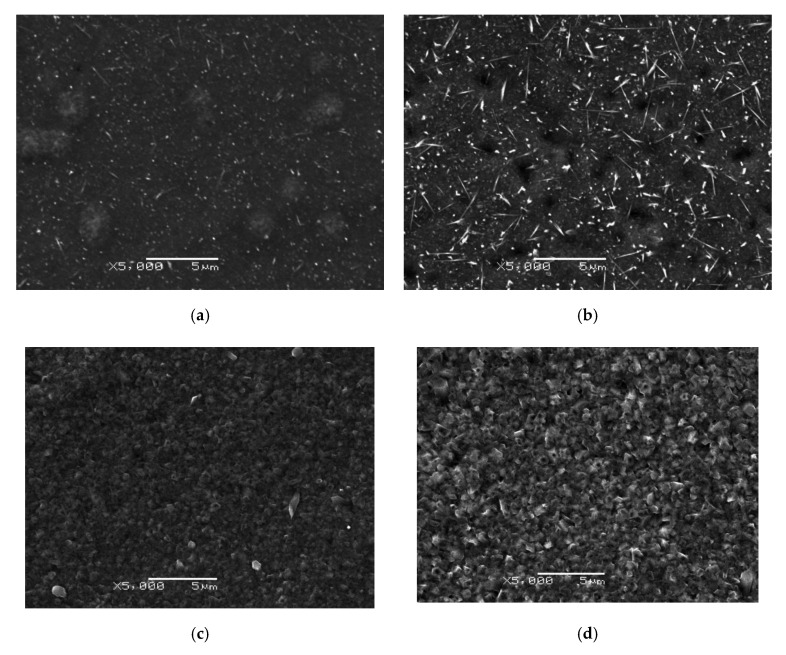

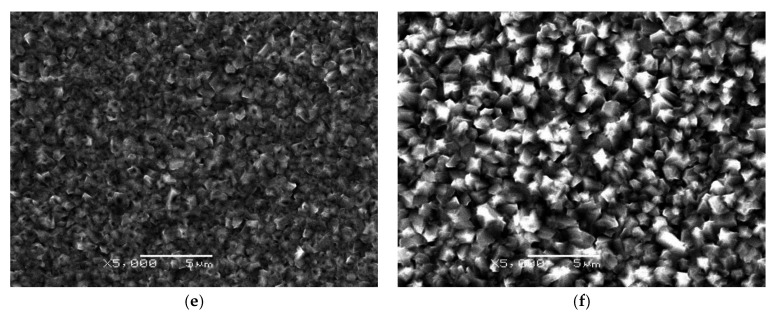
Morphology of the surface of titanium after cyclic oxidation at 600 °C ((**a**)—4 cycle/24 h, (**b**)—12 cycle/72 h), 650 °C ((**c**)—4 cycle/24 h, (**d**)—12 cycle/72 h) and 700 °C ((**e**)—4 cycle/24 h, (**f**)—12 cycle/72 h).

Microscopic images presenting the morphology of the oxides produced after cyclic oxidation of titanium Grade 2 at temperatures of 600, 650 and 700 °C are shown in Figure 6.

Microscopic observations of titanium Grade 2 after cyclic oxidation showed significant differences in the morphology of the obtained oxides, depending on the oxidation temperature and the number of cycles. No signs of scaling or chipping of the scale were observed. After oxidation at 600 °C, the obtained oxide scale was continuous and homogeneous. On the surface, oxides in the acicular system were observed. The size of the formed acicular oxides increased with an increasing number of cycles. At the same time, the oxide scale obtained after 12 oxidation cycles at the temperature of 600 °C showed the presence of craters. After cyclic oxidation at 650 °C, it was found that the surface of the specimen was covered with fine oxide particles. The examination showed that the particle size of oxides increased with the number of cycles. However, after oxidation at the temperature of 700 °C, an oxide scale was formed, which consisted of large oxide particles. The size of the oxide particles was much higher after 12 oxidation cycles. A similar surface morphology was obtained in study [31] after cyclic oxidation of the Ti-6Al-4V alloy at a temperature of 650 °C. The formation of large oxide particles is characteristic of elevated oxidation temperatures and takes place through nucleation and agglomeration of finer oxide particles [26,35]. Thus, no craters were found in the oxide scales obtained at temperatures of 650 and 700 °C.

After cyclic oxidation, a slightly different morphological structure of the oxides was obtained compared to the classical method of isothermal oxidation. In paper [33], after isothermal oxidation at a temperature of 600 °C, slightly larger particles of the obtained oxides were formed, and no presence of these particles was found in the acicular system. No craters were found in the oxide scale, either. At the same time, after cyclic oxidation at the temperature of 700 °C, the size of the oxide particles formed was much greater in comparison with the traditional method of isothermal oxidation [33].

### 3.4. Oxide Scales Thickness

**Figure 7:**
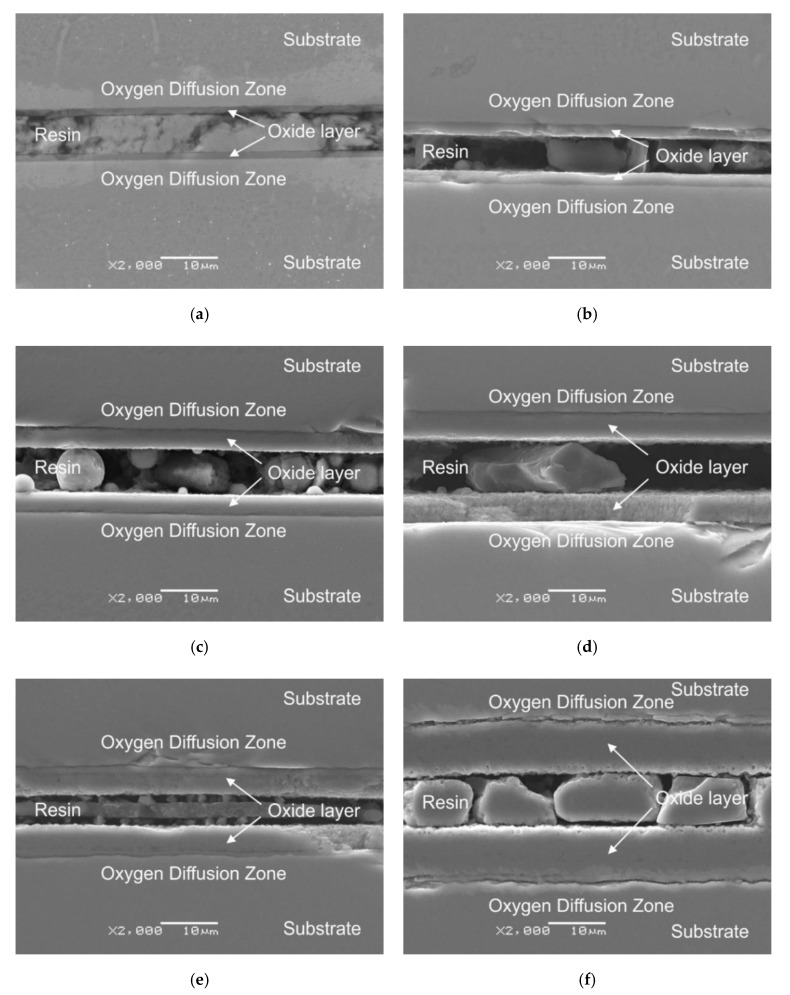
Oxide scale on a cross-section of titanium Grade 2, formed at 600 °C ((**a**)—4 cycle/24 h, (**b**)—12 cycle/72 h), 650 °C ((**c**)—4 cycle/24 h, (**d**)—12 cycle/72 h) and 700 °C ((**e**)—4 cycle/24 h, (**f**)—12 cycle/72 h).

Figure 7 shows microscopic images and the results of thickness measurements of the oxide scales after cyclic oxidation of titanium Grade 2 at 600, 650 and 700 °C in 4 and 12 cycles (24 and 72 h).

Microscopic observations of cross sections showed that the oxide scales formed during cyclic oxidation on titanium Grade 2 at 600, 650 and 700 °C were continuous and homogeneous. In addition, no presence of pores was found (Figure 7a–e). Only after oxidation at the temperature of 700 °C (12 cycles), detachment of the oxide scale from the substrate was observed, most probably as a result of the great thickness of the obtained layer and a higher influence of thermal shocks during the cyclic oxidation process. The detachment of the oxide scale from the substrate could also be influenced by the grinding process of the specimens for metallographic tests (Figure 7f). Moreover, it was stated in [36] that the cause of this type of damage may be related to the growth of large oxide grains during oxidation, which expand, thereby causing the phenomenon of compression and collisions between larger oxide grains. In this way, the bonding force between the substrate and the oxide scale is weakened, resulting in a worse adhesion of the layer to the substrate. The oxygen diffusion zone could also have a significant influence on the adhesion of the oxide scales. The deterioration of adhesion after oxidation at the temperature of 700 °C (12 cycles) may have been associated with the phenomenon of oxygen diffusion, which strongly affects the mechanical properties of titanium materials, especially the reduction of ductility and fatigue life [37]. This is confirmed by the tests of mechanical properties carried out on titanium Grade 2, which showed that an increase in oxidation temperature leads to a decrease in strength and plasticity properties [38]. According to the literature data, increasing the temperature and extending the oxidation time results in a deeper oxygen penetration in the oxygen diffusion zone [26,39]. Moreover, with the increase in temperature and oxidation time, the oxygen content in the diffusion layer increases, which results in an increase in hardness [26], and this is confirmed in the further part of the study. In paper [37] it is shown that the thickness of the obtained oxide scales and the depth of the oxygen diffusion zone are not directly proportional and differ depending on the material.

**Figure 8:**
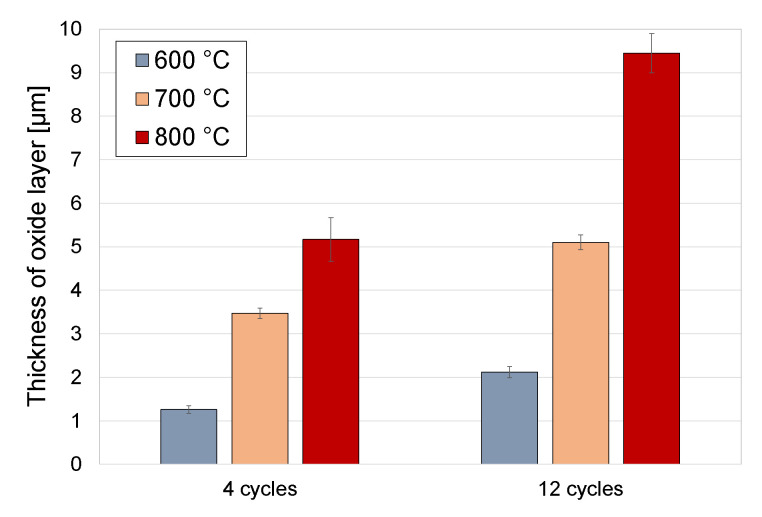
Thickness of oxide scales depending on the oxidation temperature and number of cycles.

Figure 8 shows the dependence of the average thickness of oxide scales on cyclic oxidation parameters. The tests showed a tendency for the oxide scale thickness to increase along with an increase in the temperature applied and the number of cycles. However, it was also shown in the tests that the number of oxidation cycles had less effect on the intensification of the oxidation process compared to temperature.

In the cyclic oxidation process, oxide scales of various thicknesses were obtained, depending on the oxidation conditions (temperature and number of cycles). Due to the low intensity of the oxide scale growth at 600 °C, its thickness was relatively low (1.26 and 2.12 µm, respectively). An increased number of cycles from 4 to 12 resulted in an approx. 68% increase in the oxide scale thickness. After oxidation at 650 °C, the oxide scales produced had greater thicknesses. After 4 oxidation cycles at 650 °C, a 3.47 µm thick layer was obtained, while after 12 cycles, the thickness amounted to 5.10 µm. Moreover, the studies showed that the oxide scales which formed after oxidation at 650 °C (12 cycles) and 700 °C (4 cycles) were characterised by the same thickness (approx. 5.1 µm). After 12 oxidation cycles at 700 °C, an oxide scale with the greatest thickness (9.45 µm) was obtained. The oxide scales produced on titanium Grade 2 were characterised by about 5 times higher thickness in comparison to the same conditions of cyclic oxidation of the Ti-6Al-7Nb alloy [40].

Compared to the traditional method of isothermal oxidation [41], the cyclic oxidation of titanium allowed obtaining oxide scales of much higher thickness. After cyclic oxidation at 600 °C (4 cycles/24 h), thickness of the newly formed oxides was three times higher than in the case of isothermal oxidation. An increased number of cycles at 600 °C, on the other hand, led to the formation of oxide scales by approx. 0.75 µm thicker compared to isothermal oxidation. After oxidation at the temperature of 700 °C (4 cycles), cyclic oxidation allowed obtaining oxide scales thicker by more than three times than in the case of isothermal oxidation under similar temperature and time conditions. After 12 oxidation cycles at 700 °C, the produced oxide scale was by approx. 57% thicker compared to the layer obtained through traditional thermal oxidation [41].

### 3.5. Phase Composition of the Oxide Scales

**Figure 9:**
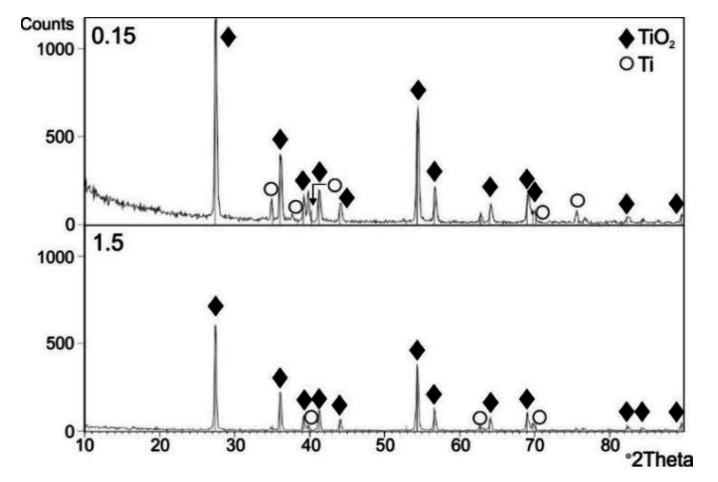
A set of diffraction patterns obtained by the GIXD method at beam incidence angles α = 0.15° and 1.5°, for a titanium Grade 2 specimen after cyclic oxidation (12 cycles) at 600 °C.

**Figure 10:**
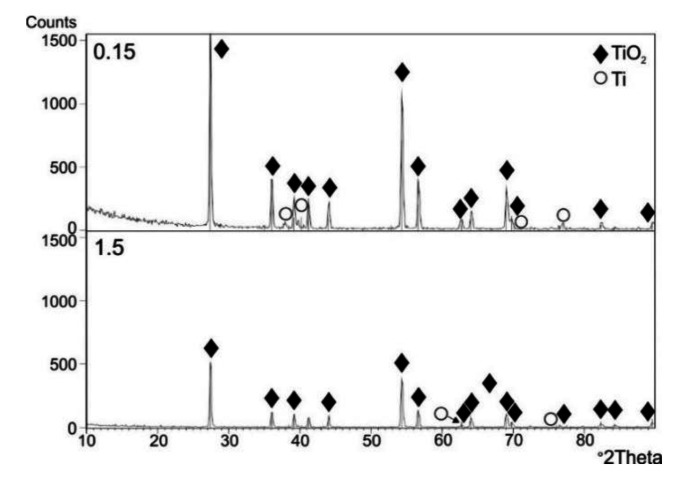
A set of diffraction patterns obtained by the GIXD method at beam incidence angles α = 0.15° and 1.5°, for a titanium Grade 2 specimen after cyclic oxidation (12 cycles) at 650 °C.

**Figure 11:**
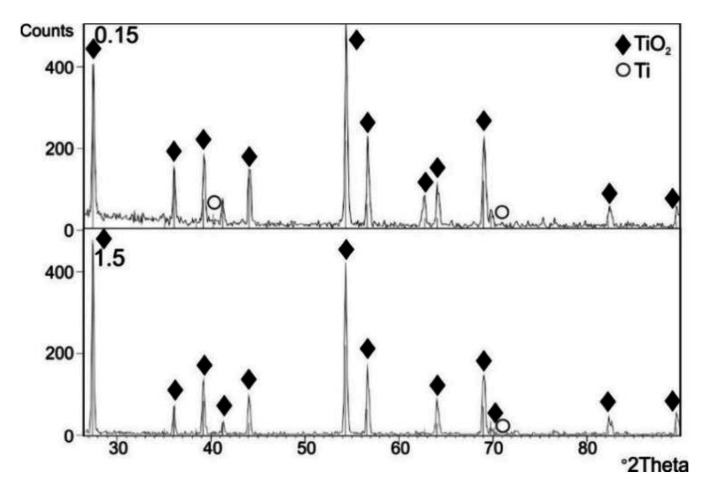
A set of diffraction patterns obtained by the GIXD method at beam incidence angles α = 0.15° and 1.5°, for a titanium Grade 2 specimen after cyclic oxidation (12 cycles) at 700 °C.

Figure 9, Figure 10 and Figure 11 present the results of the XRD examination of titanium Grade 2 subjected to cyclic oxidation at temperatures of 600, 650 and 700 °C (12 cycles).

The XRD examination after cyclic oxidation showed that the oxide scales obtained at temperatures of 600, 650 and 700 °C were composed mostly of TiO_2_, in the crystallographic form of rutile. At the same time, the presence of peaks coming from the titanium Grade 2 substrate was found. No presence of other phases was found. During isothermal oxidation with similar temperature/time parameters, peaks originating from the Ti_3_O phase were found apart from the TiO_2_ phase (rutile) [33]. At the same time, no presence of peaks coming from the titanium substrate was found.

### 3.6. Oxide Scale Hardness

**Figure 12:**
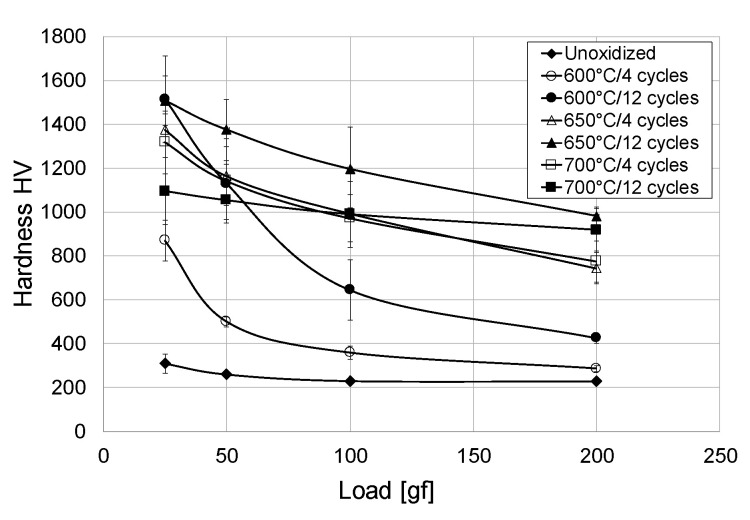
Surface hardness of oxide scales formed at different applied parameters of cyclic oxidation, depending on indenter load.

Figure 12 shows a graph with the results of surface hardness measurements of oxide scales obtained after cyclic oxidation at temperatures of 600, 650 and 700 °C, depending on the load of the indenter.

The oxide scales formed as a result of cyclic oxidation were characterised by different hardness, depending on the temperature, number of cycles and indenter load. At low loads of the indenter, the results obtained were closest to the hardness of oxide scales, while at high loads, the “composite” hardness of the oxide scale was measured simultaneously with the hardness of the oxygen diffusion zone and the substrate. After 4 oxidation cycles at a temperature of 600 °C, the scale hardness amounted to 869.6 HV (25 gf load). After 12 oxidation cycles at the same temperature the hardness increased significantly and reached the value of 1514.2 HV (load 25 gf). A further increase in the hardness of the oxides formed was obtained after oxidation at 650 °C (in particular after 4 oxidation cycles, it was found that the hardness of the layer was about 58% higher compared to that obtained at 600 °C). Increasing the number of cycles also led to an increase in hardness. However, after oxidation at the temperature of 700 °C it was found that the produced oxide scales had lower hardness compared to the oxide scales obtained at the temperature of 650 °C. This is a different result compared to the traditional method of isothermal oxidation [33]. The reason for the decrease in hardness after cyclic oxidation at the temperature of 700 °C could be a greater influence of thermal shocks and the related, slightly worse adhesion of the formed scale. It was shown in the study that hardness of the oxide scales decreased with an increasing pressure on the indenter. The hardness of the oxide scales decreased most intensely after cyclic oxidation at 600 °C (especially after 4 oxidation cycles), which indicates a low thickness of the oxide scale. The decrease in hardness was more gentle for the oxide scales obtained at temperatures of 650 and 700 °C, which was connected with their greater thickness. Similar changes in hardness with an increasing load of the indenter were described in paper [42] for the Ti-6Al-4V alloy oxidised at 400–600 °C. The decrease in hardness with increasing pressure on the indenter was related to the greater influence of the substrate on the measurement result. Furthermore, it was found that as the oxidation temperature increases, the spread of results becomes greater, as evidenced by the increasing measuring error. This may be caused by the inhomogeneity of the microstructure and increased roughness of the surface [35]. Obtaining a significant increase in surface hardness after cyclic oxidation may have a measurable effect on improvement of the weak tribological properties of titanium Grade 2 [33].

## 4. Conclusions

The study led to the following conclusions:

1. The cyclic oxidation process allowed the formation on the surface of titanium Grade 2 of oxide scales of good quality, covering the whole surface and characterised by a homogeneous structure.
2. The parameters of cyclic oxidation (temperature and number of cycles) had a significant influence on the dynamics of oxide scales growth on titanium Grade 2. The highest intensification of the process was found after oxidation at 700 °C. For this variant, the mass gain was nearly four times higher than in the case where the oxidation temperature was 600 °C.
3. The temperature of cyclic oxidation had a significant influence on the morphology of the oxide scales formed. After oxidation at 600 °C, the produced oxide scale was characterised by the presence of oxides in the acicular system. Increasing the oxidation temperature to 650 and 700 °C led to the formation of oxide scales composed of oxide particles which increased with the increasing oxidation temperature.
4. The oxide scales obtained in the cyclic oxidation process had varied thicknesses, depending on the oxidation temperature and the number of cycles. The oxidation process conducted at a temperature of 600 °C enabled the formation of oxide scales with thicknesses of 1.26 µm (4 cycles) and 2.12 µm (12 cycles). Increasing the oxidation temperature to 650 °C induced a further growth of the oxide scale thickness (to 3.47 µm and 5.10 µm, respectively). The oxide scales produced at 700 °C had the greatest thickness (5.17 and 9.45 µm, respectively).
5. XRD examination after cyclic oxidation showed presence of TiO_2_ (rutile) only in the obtained layers, regardless of the oxidation temperature. At the same time, presence of peaks coming from the titanium Grade 2 substrate was found. No presence of other phases was found.
6. The cyclic oxidation process contributed to an increase in the surface hardness of titanium Grade 2. The oxide scales which formed at 600 and 650 °C had the greatest hardness (approx. 1500 HV). After oxidation at 700 °C, a considerable decrease in hardness was found (by approx. 400 HV).
7. Cyclic oxidation, in comparison with the traditional isothermal oxidation method, is characterised by higher intensity, which allows obtaining oxide scales characterised by higher thickness. This, in turn, may positively contribute to the improvement of functional properties of titanium.

## Figures and Tables

**Table 1:** Chemical composition of titanium Grade 2.

Material	Component Content, wt.-%							
	C	Fe	H	N	O	Al	Nb	Ti
TiGr2	0.008	0.13	0.0019	0.010	0.18	-	-	the rest

**Table 2:** Titanium Grade 2 grain size parameters.

ASTM Grain Size Number	11
Mean Grain Area [µm^2^]	63
Total Number of Grains	822
Analyzed Area [µm^2^]	52,988

**Table 3:** Values of the parabolic rate constant for oxidation, K_p_.

	Temperature [°C]		
	600	650	700
K_p_ [(mg^2^/cm^4^s)] × 10^−8^	229	1120	4189
